# Roof structure of shallow coal seam group mining in Western China

**DOI:** 10.1371/journal.pone.0255047

**Published:** 2021-08-12

**Authors:** Jian Cao, Qingxiang Huang

**Affiliations:** 1 Institute of Mining Engineering, Inner Mongolia University of Science and Technology, Baotou, Inner Mongolia, China; 2 School of Energy, Xi`an University of Science and Technology, Xi`an, Shaanxi, China; China University of Mining and Technology, CHINA

## Abstract

In order to realize roof control of shallow coal seam group mining in Western China, combining with engineering statistics, physical simulation and theoretical analysis, the roof weighting characteristics during lower coal seam mining were revealed, and the classification of shallow coal seam group was proposed. Based on this, mechanical models of roof structure were set up, and the calculation method of support resistance was determined. The results show that the roof weighting is closely related to the interburden thickness and the mining height of lower coal seam, considering the ratio of interburden thickness to the mining height, as well as the key stratum structure, the classification of shallow coal seam group was put forward. The first type is shallow coal seam group with no key stratum (SCSG-No), its roof pressure is mainly affected by caving roof of upper coal seam, and the interburden roof forms slanting pillar-beam structure. The second type is shallow coal seam group with single key stratum (SCSG-S), interburden roof represents step voussoir beam structure. The third type is shallow coal seam group with double key strata (SCSG-D), interburden roof can form double key strata structure, the lower key stratum forms slanting step voussoir beam structure, while the upper key stratum forms voussoir beam structure, besides, longwall face represents large—small periodic weighting. Through establishing the roof structure models, the calculation formulas of support resistance were determined, it can provide basis for roof control and promote safe mining in Western China.

## Introduction

Shallow coal seams in Jurassic Coalfield are generally characterized by shallow depth (less than 300 m), small interburden (strata between upper and lower coal seam) thickness (less than 45 m), and 2–3 coal seams are mainly mined, consequently, it belongs to shallow coal seam group mining.

Recently, the upper coal seam in Jurassic Coalfield was already mined out, and mainly mines lower coal seam. During lower coal seam mining, roof weighting is complex, and supports damage caused by dynamic load is more frequent. In 2017, the number of people who died because of coal mine disasters is 357 in China, and 25% of those disasters are roof accidents. Therefore, it is important to reveal roof structure and determine the reasonable support resistance.

Since 1990s, scholars have carried out a systematic study on shallow single coal seam mining. Roof structure theory, represented by the definition of shallow coal seam and “step voussoir beam structure”, had been put forward by Huang [1, 2]. Zhang and Wang. [3] studied the structure and stability of overlying strata in shallow coal seam mining. Tian et al. [4] revealed the roof structure of gob-side entry retaining in shallow coal seam. Wang et al. [5] analyzed the catastrophe evolution of the stratified roof in shallow coal seams. Through physical simulation, Ding and Liu. [6] studied the movement law of overlying strata in shallow shortwall seam mining. Wang et al. [7] obtained the instability mechanism of roof in sandy gullies conditions. In addition, the roof control theory of large mining height in shallow coal seam was also proposed [8–11]. Overall, the roof structure and its control theory in shallow single seam mining had already been established.

**Fig 1:**
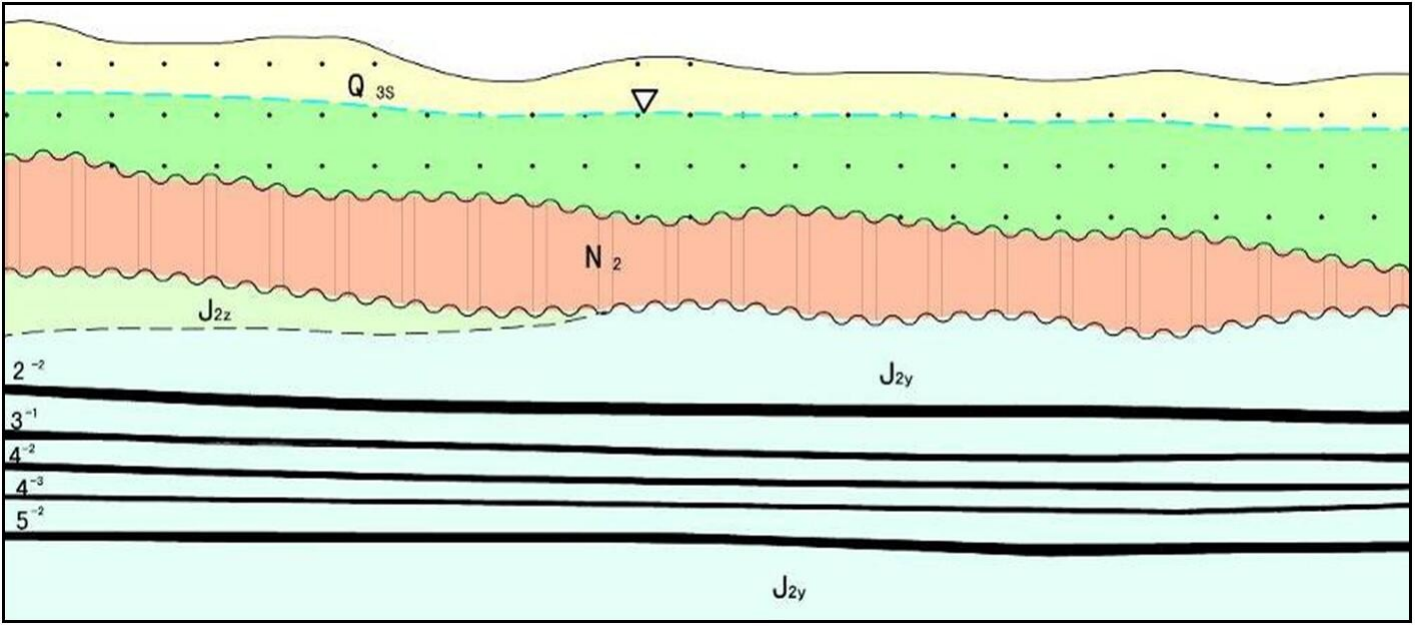
Shallow coal seam group in Jurassic Coalfield.

In recent years, scholars have carried out studies related to shallow coal seam group mining, which mainly can be divided into two aspects. One is the roof control method of abnormal roof pressure when mining under overlying coal pillar [12–15], another is the roadway support method during lower coal seam mining [16–21]. However, studies related to the roof structure are still not systematic enough, Yu and Gong [22, 23] established the breaking roof structure model of lower seam mining. Zhu et al. [24] proposed the “loosen-blocky” roof structure model. Yang and Xie [25, 26] studied the roof structure under room-and-pillar goaf, the “main roof-coal pillar-immediate roof-supports” roof structure system was established. Studies above are beneficial to this paper, whereas, based on mining conditions in Jurassic Coalfield (Fig 1), the thickness of interburden and coal seams varies in a wide range, which causes unclear roof weighting characteristics, consequently roof structure and support resistance are still to be further studied.

This paper takes shallow coal seam group mining in Western China as background, combining with physical simulation, theoretical analysis and engineering practice, roof weighting characteristics were revealed, and the classification of shallow coal seam group was put forward, in addition, roof structure models were established, and calculation methods of support resistance were given. In the long run, it can provide basis for roof control in Western China.

## Classification of shallow coal seam group

### Roof weighting characteristics

In Jurassic Coalfield, although mining height, burial depth and interburden thickness are different, there still have connections in dip angle of coal seams, lithology and so on, therefore, summarizing roof weighting characteristics has important significance. Engineering statistics of Jurassic Coalfield are shown in Table 1, based on the statistics data, roof weighting characteristics and influence factors were analyzed.

**Table 1:** Engineering statistics of shallow coal seam group mining in Jurassic Coalfield.

Coal mine	Mining height of upper seam(m)	Lower seam							
		dip angle (°)	Mining height (m)	Interburden thickness (m)	Ratio of interburden thickness to mining height	Number of key strata	Roof weighting characteristics		
							Periodic weighting interval(m)	Support resistance (kN)	Dynamic load coefficient
Nalinmiao LW6104	2.1	1–3	2.6	2.9	1.2	0	12	4500	1.27
Shigetai LW12103	2.1	2	2.8	6.0	2.1	0	10	8568	1.35
Daliuta LW12306	2.67	<3	4.3	20	4.1	1	9	10693	1.62
				15	3.5	1	9	10781	1.5
Daliuta LW21305	3.78	<3	4.3	19	4.4	1	10	10887	1.72
Daliuta LW12312	3.5	<3	4.7	20	4.3	1	12	12868	1.33
Daliuta LW22303	3.52	<3	4.5	28	6.2	1	12	12007	1.33
Bulianta LW32301	6.0	1–3	6.1	32	5.2	1	17	11517	1.53
Bulianta LW22306	5.4	1–3	6.8	35	5.1	2	Small, 13 Large, 37	16900 19700	1.6
Ningtiaota LWN1200	1.72	1–3	5.9	39	6.6	2	Small, 8 Large, 24	12515 13872	1.78

#### Mining height

Generally, mining conditions are characterized by shallow buried, small dip angle and small interburden thickness. According to Table 1, during lower coal seam mining, as the mining height of upper coal seam increases, the support resistance and periodic weighting interval increase by 1.8% and 7.6% respectively, whereas they increase by 18.2% and 21.2% with the increase of mining height of lower coal seam. Therefore, mining height of lower coal seam has more obvious effect on the roof weighting.

#### Key strata and ratio of interburden thickness to mining height

When the interburden thickness is the same, as the mining height of lower seam changes, the roof caving characteristics are revealed. As the mining height increases, the development height of caving zone increases, meanwhile, the key strata structures are also different, it can form “double key strata”, “single key stratum” and “no key stratum” respectively. Therefore, interburden thickness and mining height can affect the key strata structure, and further affect the roof weighting characteristics.

Based on the analysis above, ratio of interburden thickness to the mining height is defined, written as *G*, which can be used as an index to obtain the classification of shallow coal seam group, based on *G* and the number of key strata, there are three cases during shallow coal seam group mining:

1. *Interburden with no key stratum*. As shown in Fig 2, based on engineering experience, bulking coefficient of immediate roof is 1.3, according to classical theory, the thickness of immediate roof fillings full of goaf is 3.3 times of the mining height. It means that when *G* is less than 3.3, interburden usually shows as immediate roof and can not form key stratum. Due to the mining height of lower coal seam in Jurassic Coalfield usually is 3–7 m, consequently the interburden thickness that can not form key stratum is usually less than 10–22 m. As shown in Table 1, when *G* is less than 3.3, there is no key stratum in interburden, dynamic load coefficient is relatively smaller.

**Fig 2:**
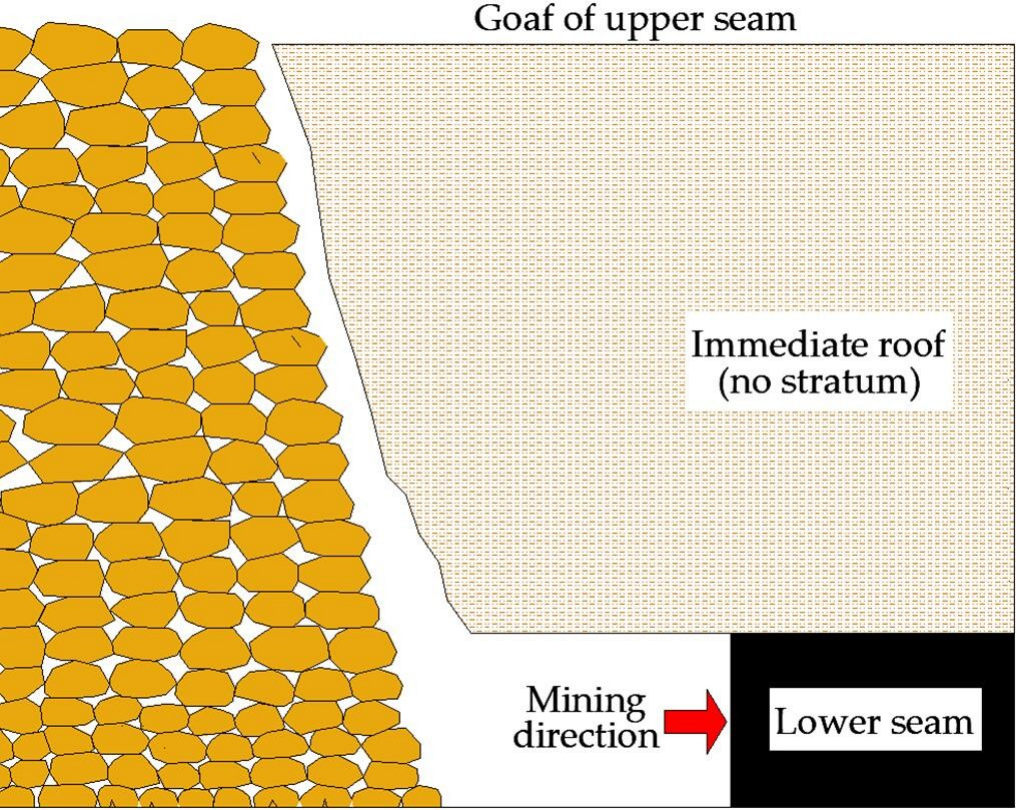
Interburden with no key stratum.
2. *Interburden with single key stratum*. As shown in Fig 3, according to engineering statistics and physical simulation, periodic weighting interval of the key stratum is generally 8–12 m in Jurassic Coalfield, and the lumpiness (ratio of key stratum thickness to periodic weighting interval) of key stratum is about 1, consequently the thickness of key stratum is about 8–12 m, it is approximately 1–4 times of the mining height. If the thickness of immediate roof is estimated with 3.3 times of the mining height, it could be calculated that the interburden thickness with single key stratum is about 4.3–7.3 ((1–4)+3.3) times of the mining height, and *G* is 4.3–7.3 (As the mining height increases, *G* decreases), consequently the interburden thickness is about 15–31 m, the result is in good agreement with Table 1 (Daliuta coal mine and LW32301 in Bulianta coal mine).

**Fig 3:**
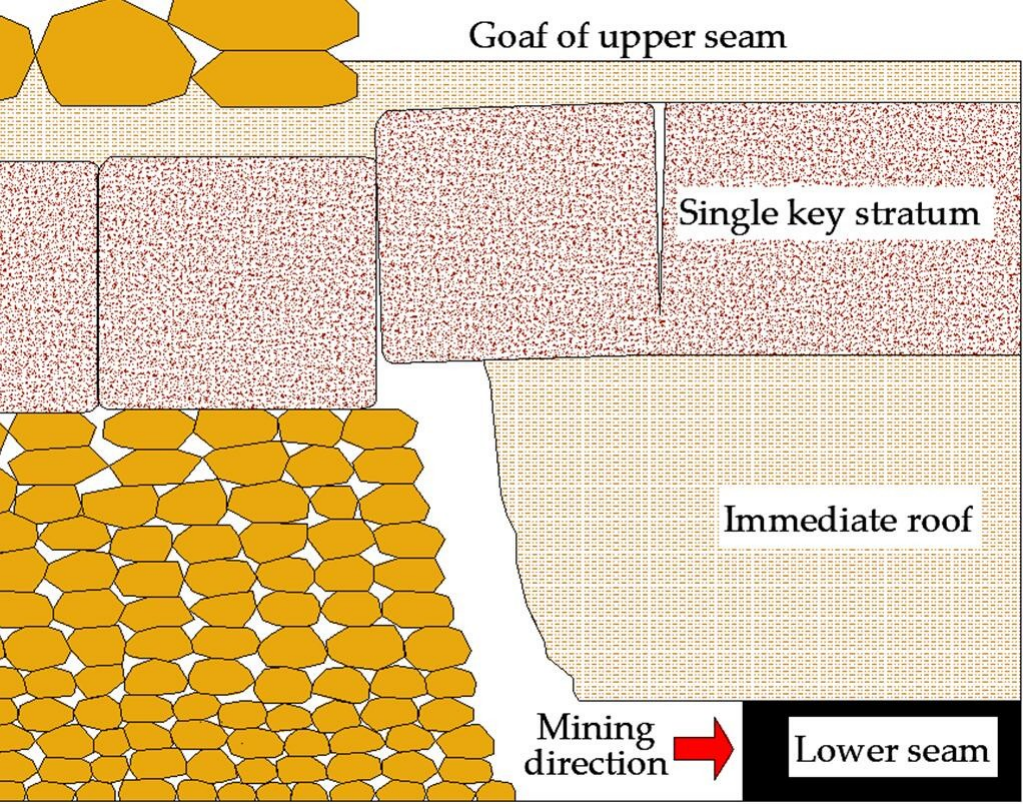
Interburden with single key stratum.
3. *Interburden with double key strata*. As shown in Fig 4, If the thickness of immediate roof is calculated with 3.3 times of the mining height, in addition, both of the key strata thickness is 1–4 times of the mining height, it could be calculated that the interburden thickness with double key strata should be larger than 5.3–11.3 times of the mining height, *G* should be larger than 5.3–11.3 (As the mining height increases, *G* decreases). For instance, if the mining height is 3 m and *G* is 11.3, the interburden thickness with double key strata should be larger than 33.9 m, another instance, if the mining height is 7 m and *G* is 5.3, the interburden thickness with double key strata should be larger than 37 m, analysis above is also verified in Table 1.

**Fig 4:**
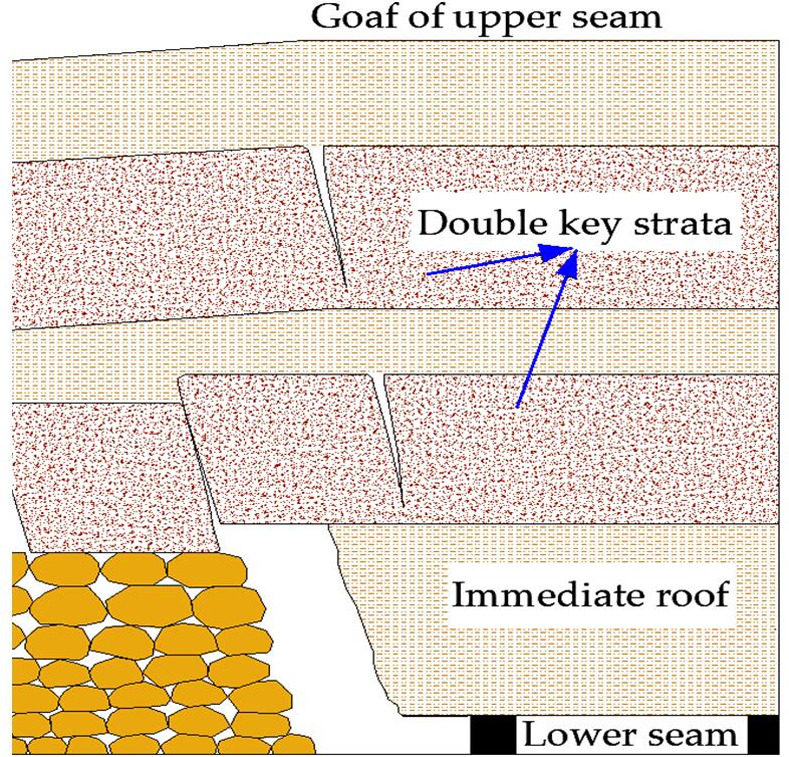
Interburden with double key strata.

### Classification of shallow coal seam group

According to the engineering statistics and theoretical analysis above, based on the key strata in interburden and the index *G*, shallow coal seam group can be divided into three types below:

1. The first type is shallow coal seam group with no key stratum (SCSG-No). Interburden thickness is generally less than 3.3 times of the mining height, *G* is less than 3.3. Interburden shows as immediate roof, and it generally can not form key stratum, the dynamic load coefficient is small.
2. The second type is shallow coal seam group with single key stratum (SCSG-S). Interburden thickness is 4.3–7.3 times of the mining height, and *G* is about 4.3–7.3. Roof shows as step voussoir beam structure, and the dynamic load coefficient is larger.
3. The third type is shallow coal seam group with double key strata (SCSG-D). Interburden thickness is larger relatively, and it can form double key strata. Its thickness is approximately 5.3–11.3 times of the mining height, *G* is about 5.3–11.3. According to Table 1, longwall face acts as large—small periodic weighting.

## Physical simulation of shallow coal seam group mining

### Physical simulation of SCSG-No

#### Engineering background

**Fig 5:**
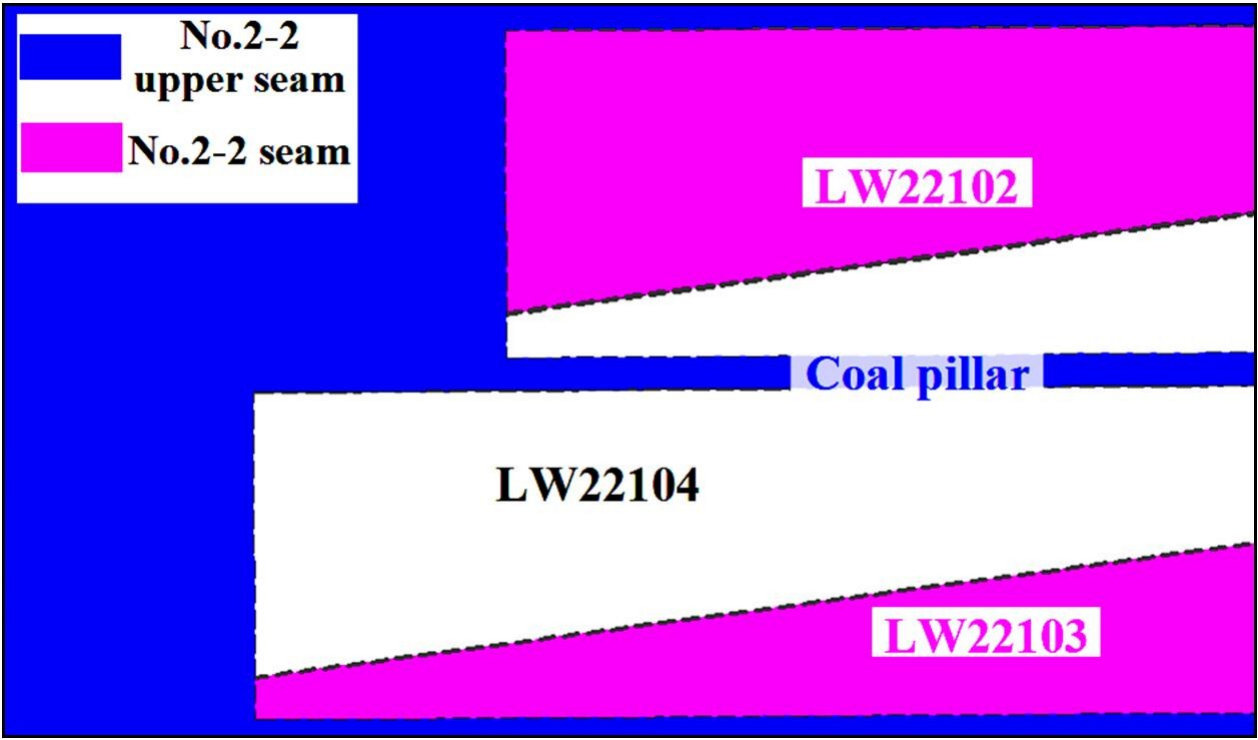
Longwall faces in Huoluowan coal mine.

Huoluowan coal mine mainly mines No.2-2 upper seam and No.2-2 seam, LW22104 is located at No.2-2 seam, LW22102 and LW22013 are located at No.2-2 upper seam which has been mined out, Fig 5 shows the layout of the longwall faces.

**Fig 6:**
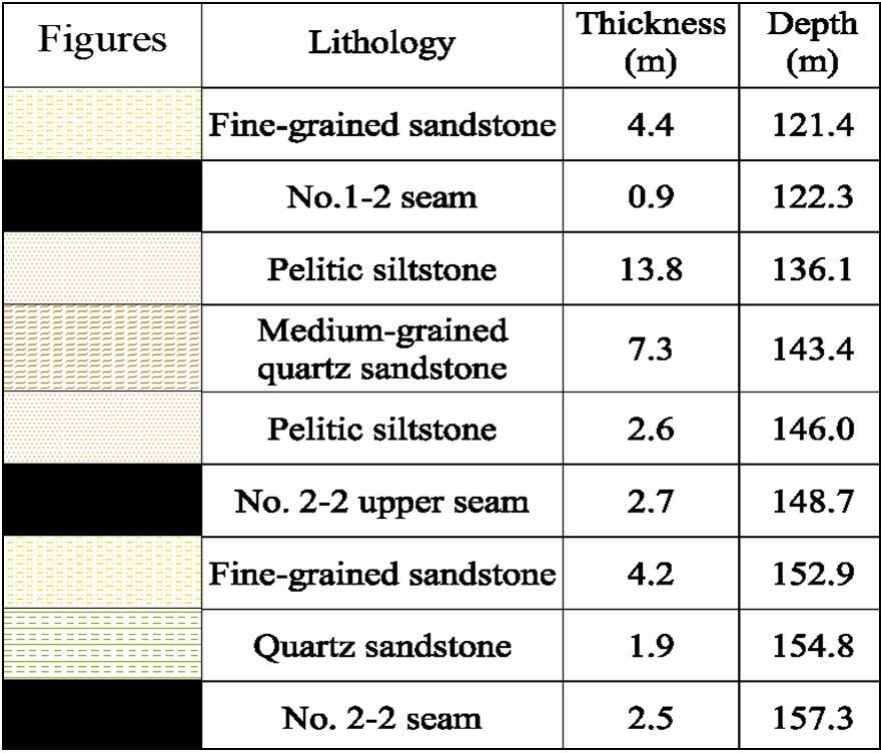
Lithology of Huoluowan coal mine.

Mining height of No.2-2 upper seam is 2.7 m, and the mining height of No.2-2 seam is 2.46m, LW22104 mines 157 m deep and dips 1–3°, the interburden thickness is 6 m on average, *G* is 2.44 (6 m/2.46 m), therefore, it belongs to SCSG-No. Based on drillhole ZK1511, lithology of Huoluowan coal mine is listed in Fig 6.

#### Physical simulation

**Fig 7:**
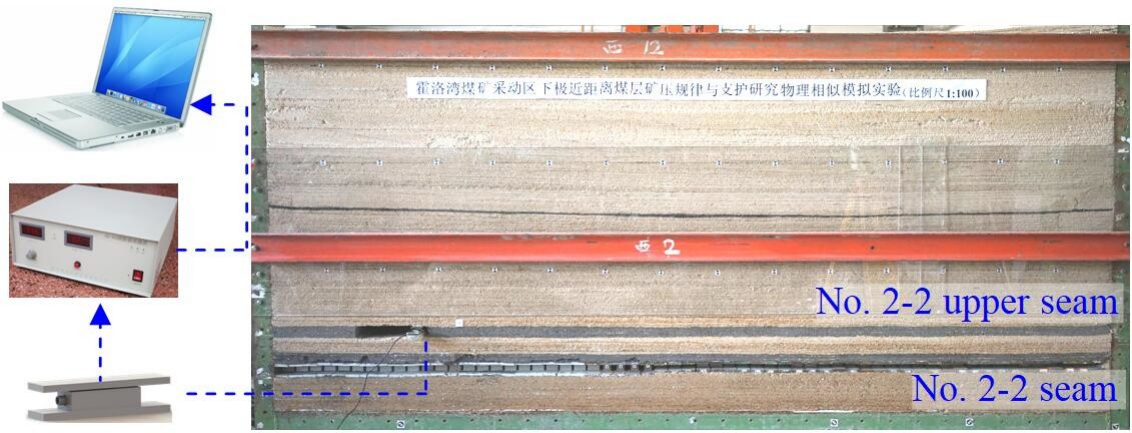
Physical model of Huoluowan coal mine.

In order to explore the roof structure of SCSG-No mining, and reveal the mechanism of roof weighting, physical simulation model was built with dimensions of 3 m long × 0.2 m wide × 1.2 m high. The major similarity coefficients were chosen as follows: geometric ratio *α*_*l*_ is 1:100, bulk density ratio *α*_*γ*_ is 1:1.5, strength ratio *α*_*R*_ is 1:150. Considering the lithology and raw material property, during model set up, sand was used as the aggregates, gypsum and calcium carbonate were adopted as cementitious materials, mica powder was used to simulate tectonic fissure, and the physical simulation model is shown in Fig 7.

The CL-YB-141 force test sensors were applied to monitor the support load during lower coal seam mining, in addition, digital camera was used to record the deformation and fracture of overburden. Firstly, the No. 2–2 upper seam was excavated, and then the LW22104 in No. 2–2 seam was mined.

#### Roof structure characteristics

**Fig 8:**
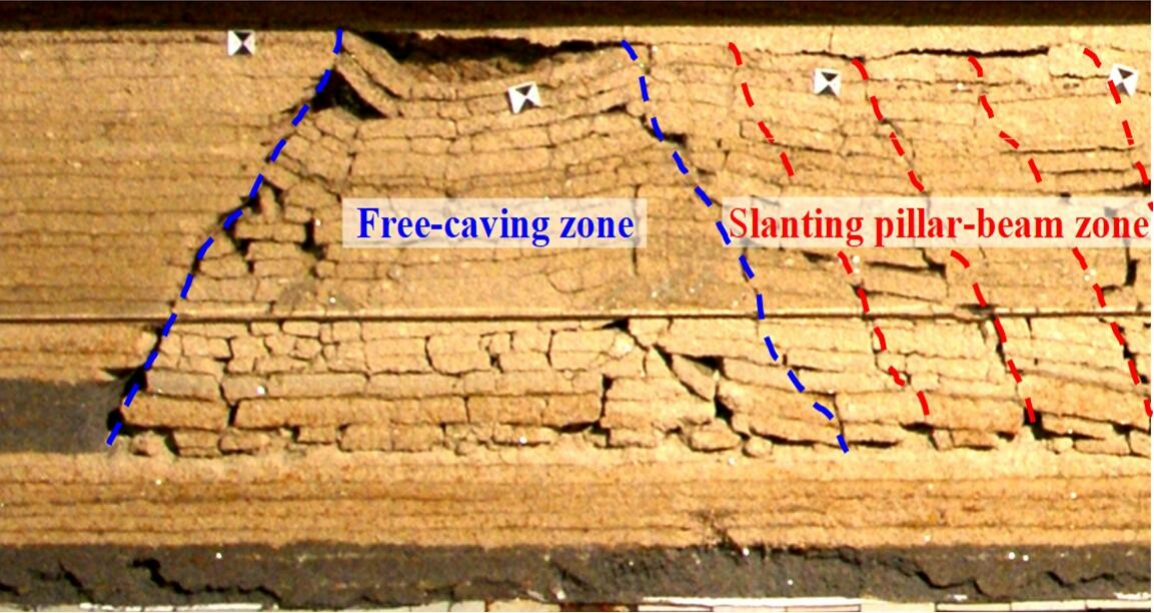
Roof caving characteristics of upper seam mining in Huoluowan coal mine.

According to the physical simulation, after upper coal seam mining, the caving roof mainly forms two zones (Fig 8), one is “free-caving zone” which the roof can not form articulated beam structure, another is “slanting pillar-beam zone”. Voussoir beam is 2 times the length of slanting pillar-beam.

**Fig 9:**
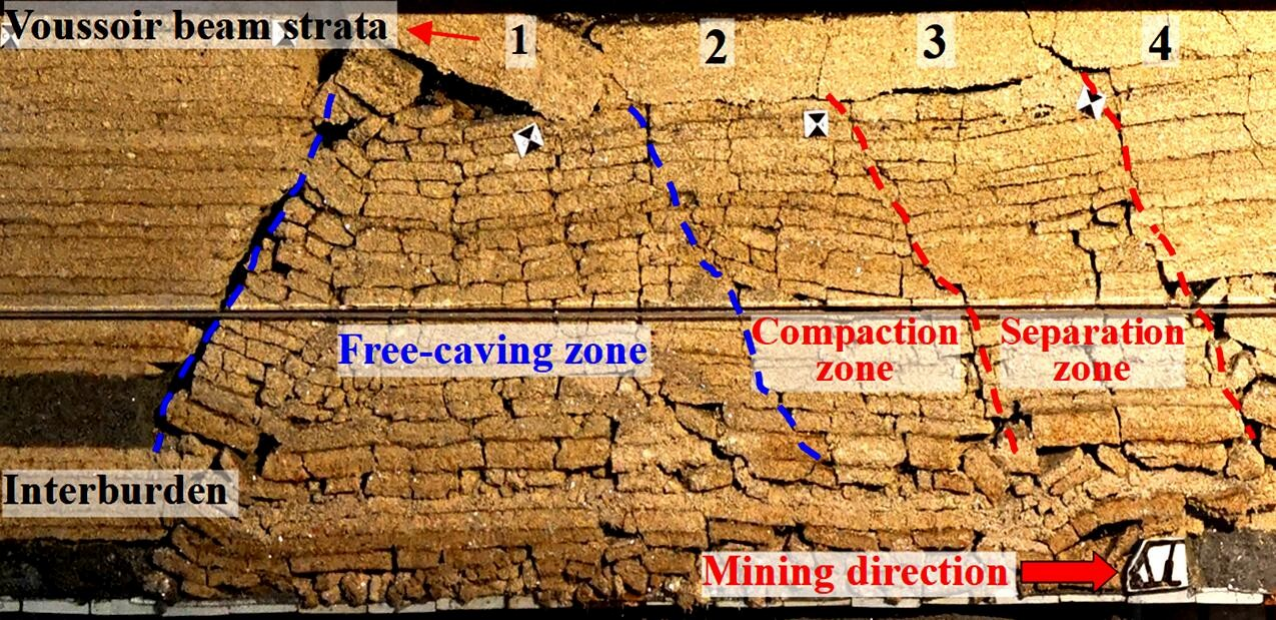
Roof structure of SCSG-No mining by physical simulation.

**Fig 10:**
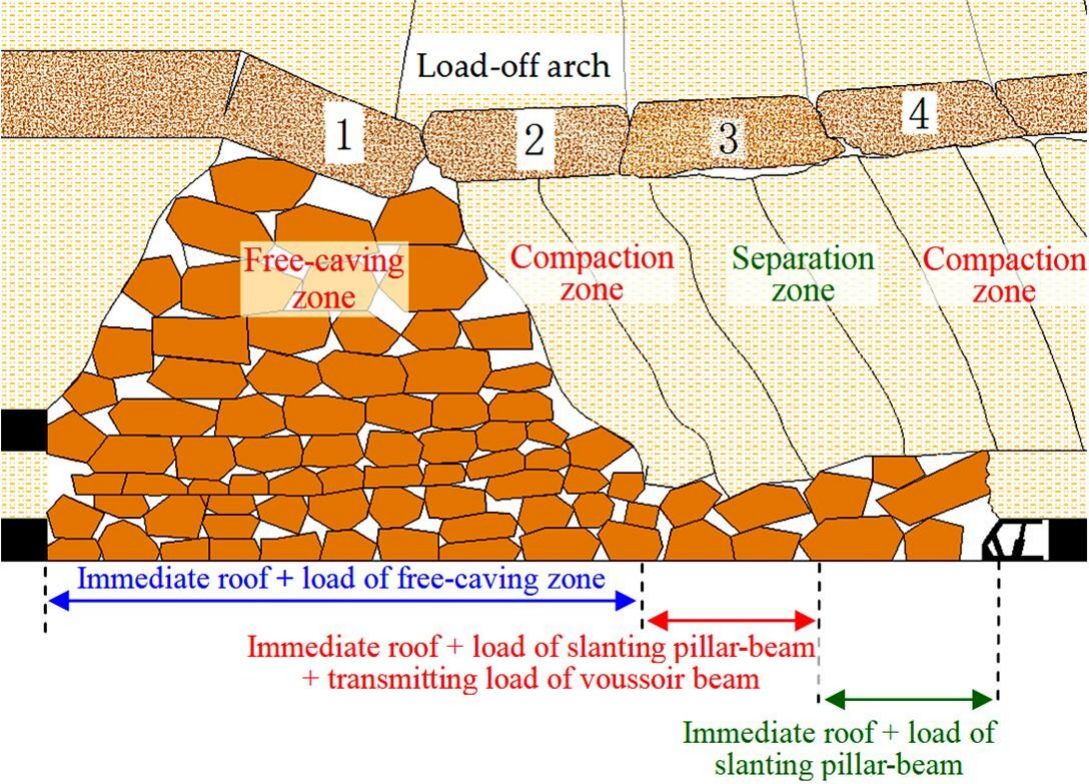
Sketch of roof structure.

During lower coal seam mining, interburden acts as immediate roof, roof caving characteristics are revealed. Caving roof can be divided into three zones, including free-caving zone, compaction zone and separation zone (Fig 9), compaction zone and separation zone are alternate periodically. The load constitution of support is shown in Fig 10, the characteristics of three zones are as follows:

1. Free-caving zone: Roof can not form key stratum and caves in time as the longwall face advances. The load of support is the weight of immediate roof and free-caving zone roof.
2. Compaction zone: When mining under the voussoir beam blocks 2 and 4 (Fig 9), the upper voussoir beam block can transmit load to lower slanting pillar-beam. The load of support mainly is the weight of immediate roof, load of slanting pillar-beam and the transmitting load of voussoir beam, it results in large periodic weighting.
3. Separation zone: When mining under the voussoir beam block 3 (Fig 9), there is interlayer separation between block 3 and lower slanting pillar-beam. The load of support is the weight of immediate roof and the load of slanting pillar-beam, it results in small periodic weighting.

**Fig 11:**
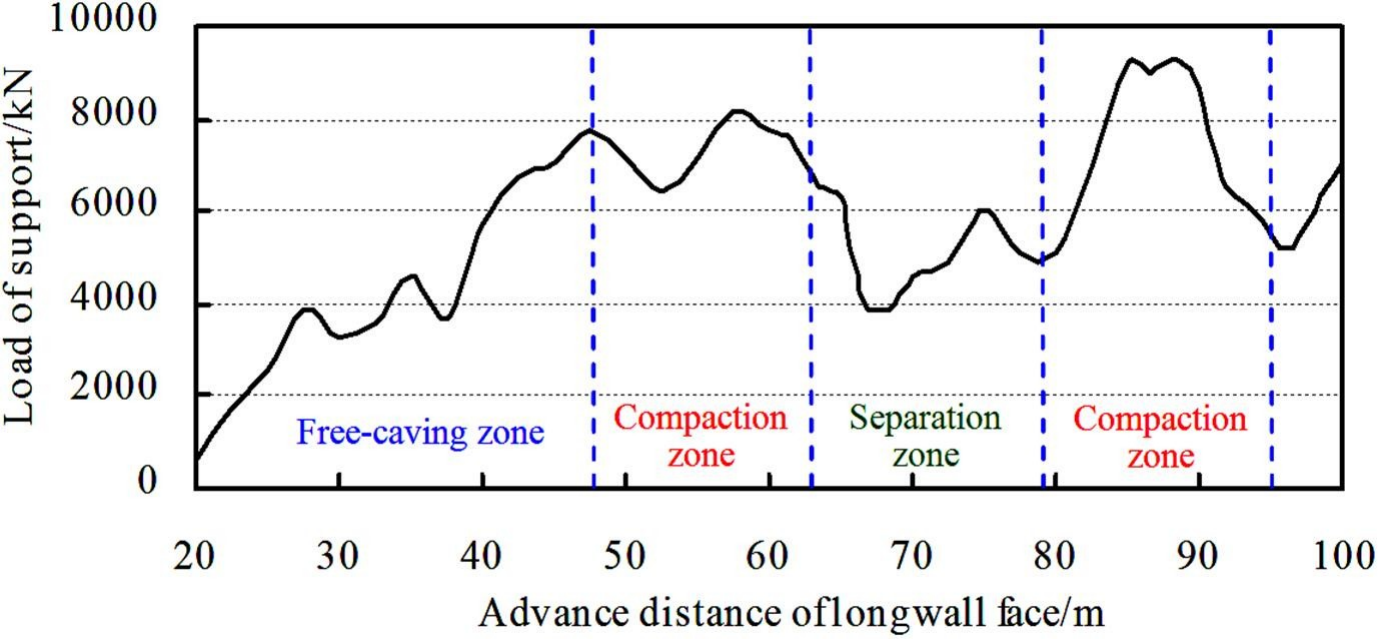
Load of support vs. longwall face advances.

Obviously, when mining under compaction zone, the load of support is the largest, and it is also verified by the physical simulation (Fig 11). Overall, taking consideration of the most dangerous situation of roof control, the support resistance should be calculated by compaction zone.

### Physical simulation of SCSG-S

#### Engineering background

**Fig 12:**
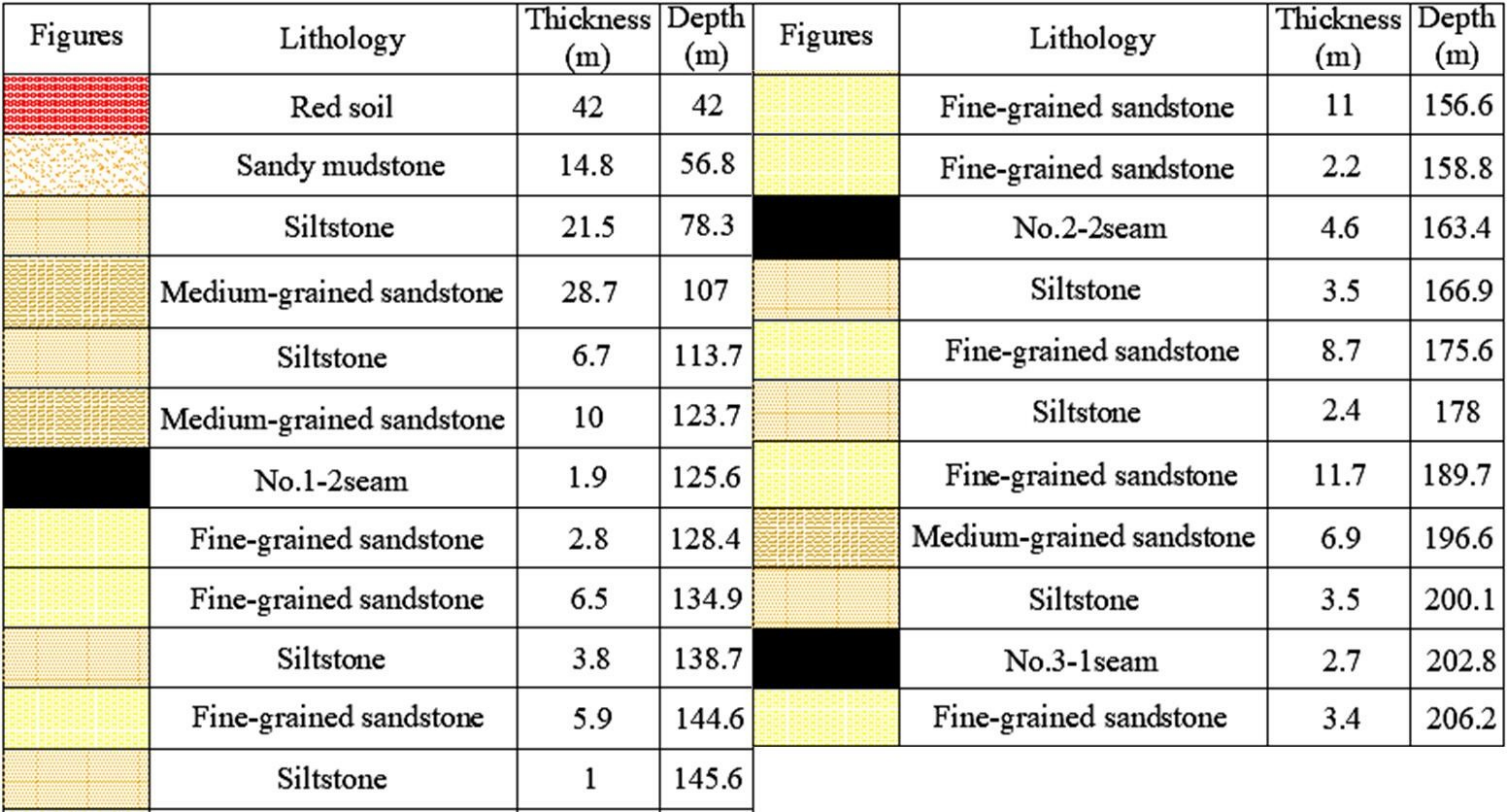
Lithology of Ningtiaota coal mine.

The No. 1–2 seam, No. 2–2 seam and No. 3–1 seam are main coal seams in Ningtiaota coal mine. They dip 1° on average, the No. 1–2 seam mines—110 m deep—is 1.89 m thick on average, and width of longwall face is 245 m. The No. 2–2 seam is 5 m thick on average, interburden thickness is 33.4 m and *G* is 6.6, it belongs to SCSG-S. The lithology is listed in Fig 12.

#### Experimental design

The physical simulation model was built with dimensions of 5 m long × 0.2 m wide × 1.03 m high. Geometric ratio *α*_*l*_ is 1:200, and time ratio *α*_*t*_ is 0.071. The raw materials of rock strata were consistent with experiment above-mentioned, as for red soil, it was found that sand and bentonite was the best aggregates, and silicone oil was the best cementitious material [27].

**Fig 13:**
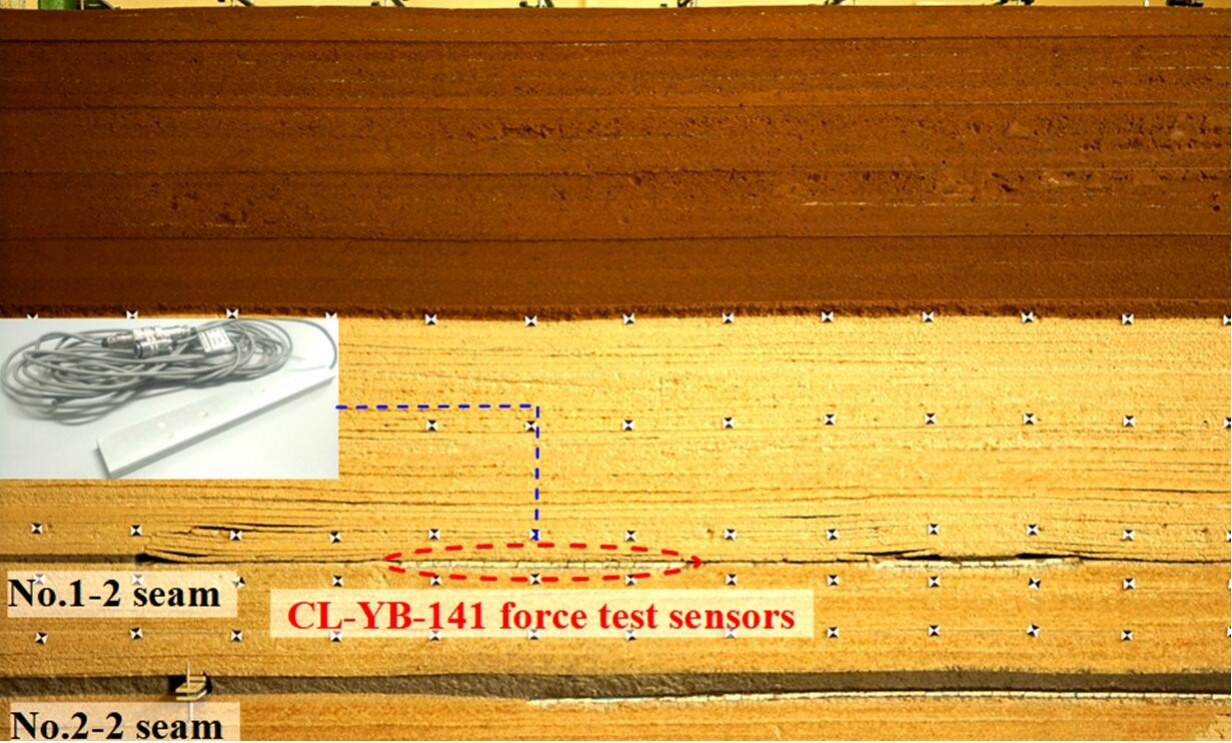
Load monitoring in physical simulation.

Firstly, the No. 1–2 seam was excavated, and then the lower No. 2–2 seam was mined. The force test sensor was applied to monitor the support load during mining, in addition, in order to obtain the transmitting load of upper goaf, the CL-YB-152 force test sensors with dimensions of 0.2 m long × 0.025 m wide × 0.01 m high, were applied at the top of the interburden (Fig 13).

#### Roof structure characteristics

**Fig 14:**
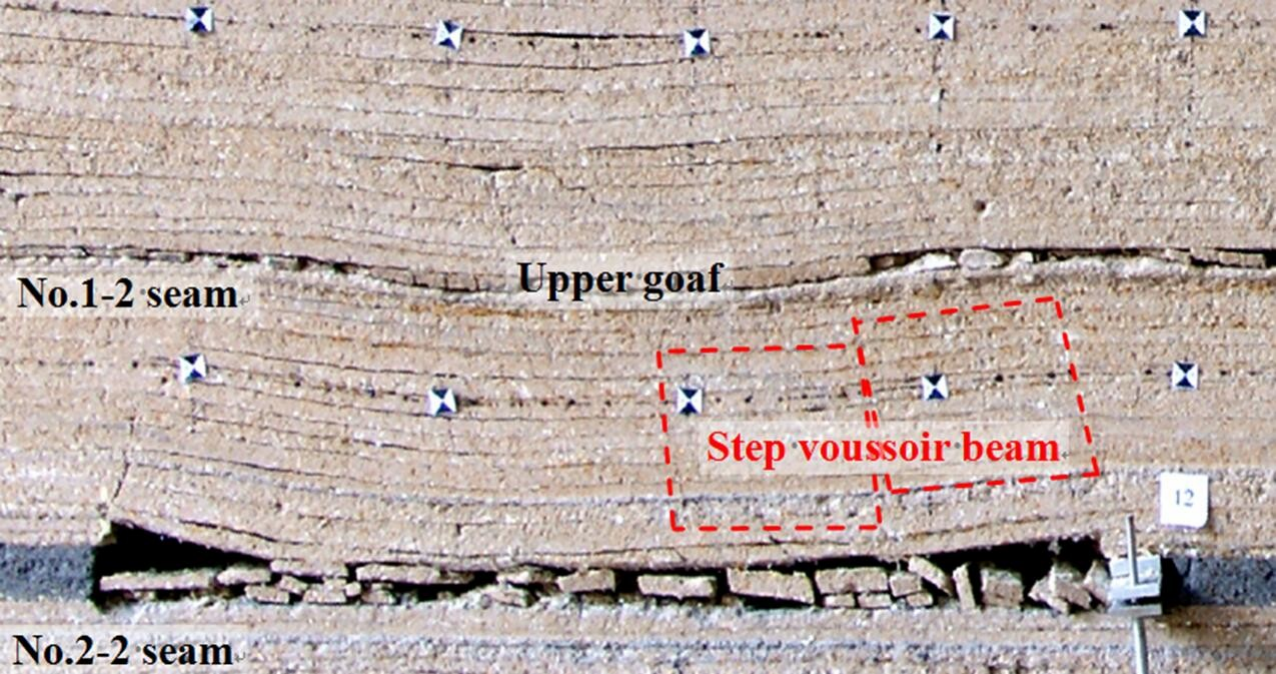
Roof structure of SCSG-S mining.

**Fig 15:**
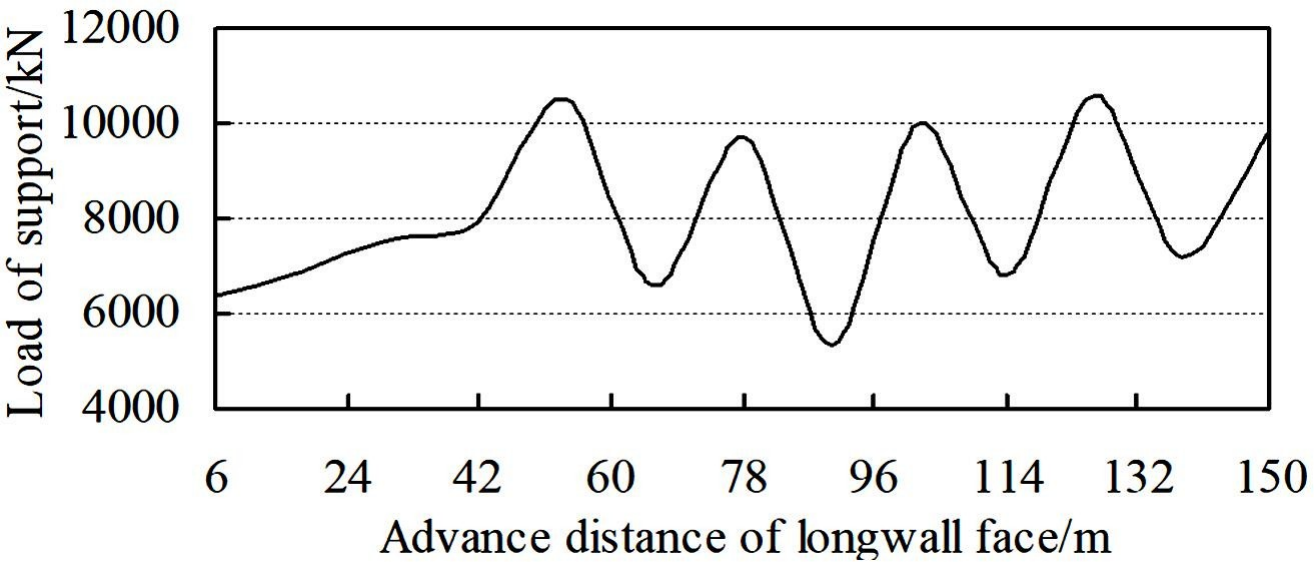
Load of support vs. longwall face advances.

As the lower coal seam mining, firstly, immediate roof fractures and caves, then, the single key stratum fractures periodically and forms step voussoir beam, the key stratum mainly bears the transmitting load of upper goaf (Fig 14). Fig 15 shows the load of support with the longwall face advances, working resistance during periodic weighting is 9695.5–10592.7 kN, based on engineering practice of LWN1206, the maximum working resistance was 10794 kN, consequently physical simulation and engineering practice are basically consistent.

### Physical simulation of SCSG-D

The No. 2–2 seam in Ningtiaota coal mine is 5 m thick on average, the No. 3–1 seam is 2.8 m thick, interburden thickness is 33.7 m and *G* is 12. therefore, it belongs to SCSG-D. The geometric ratio is 1:200.

**Fig 16:**
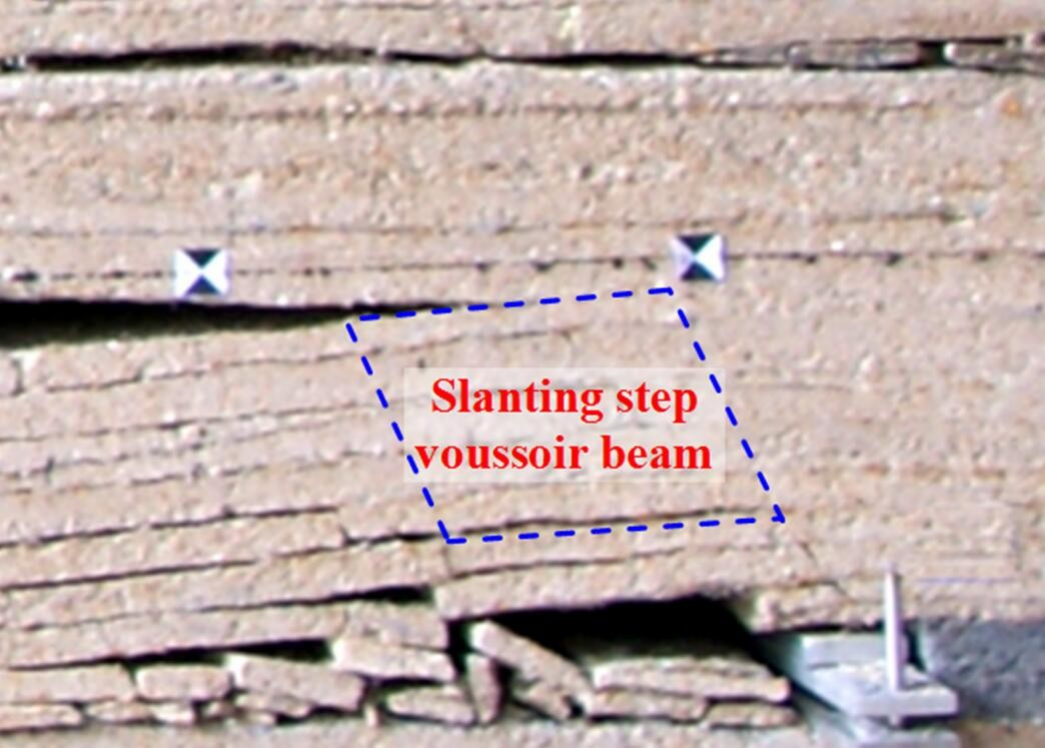
Roof structure of small periodic weighting.

**Fig 17:**
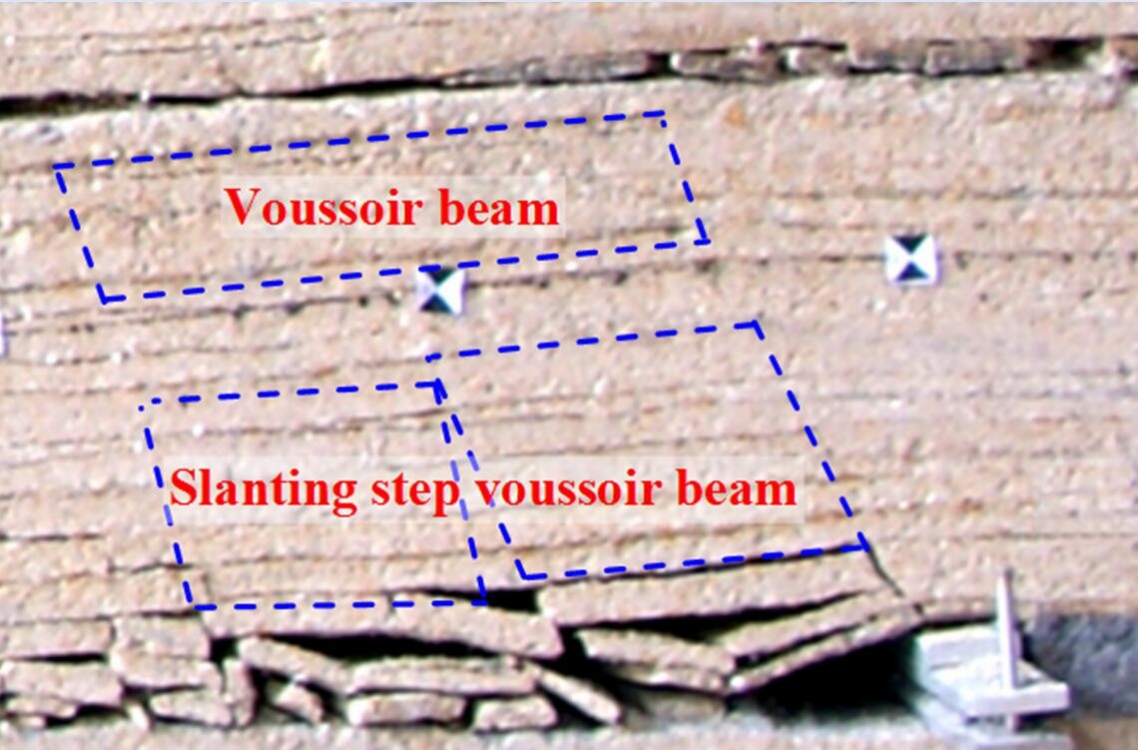
Roof structure of large periodic weighting.

As the longwall face advances, the lower key stratum fractures firstly, roof pressure is less intense (Fig 16), as the longwall face continues to advance, the double key strata fracture simultaneously, which results in intense roof pressure (Fig 17).

## Roof structure models of shallow coal seam group

### Roof structure model of SCSG-No mining

#### Mechanical model of roof structure

**Fig 18:**
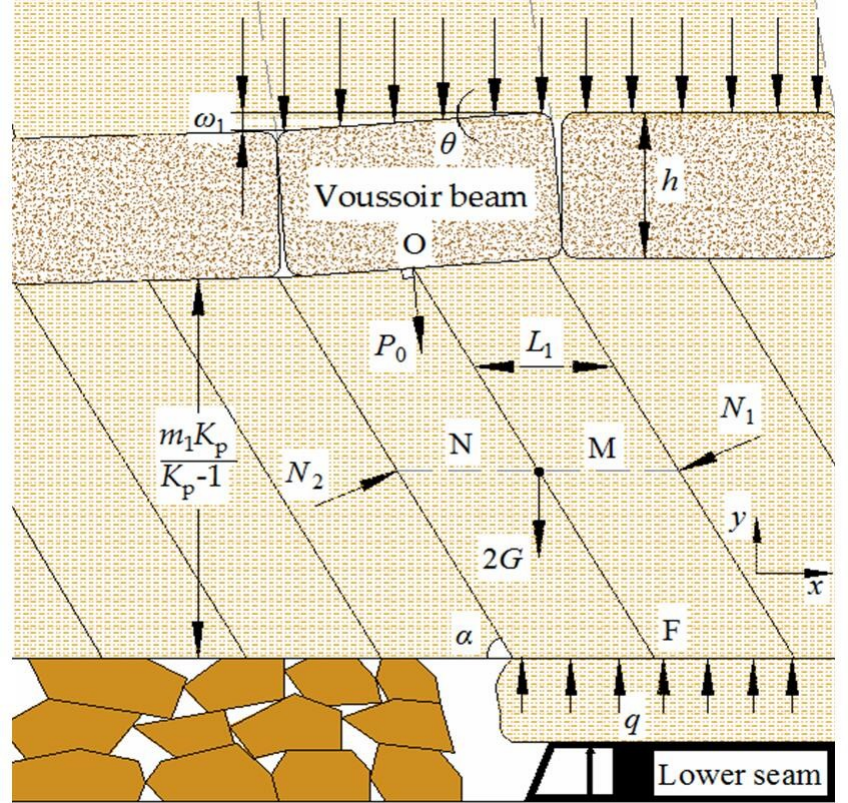
Roof structure model of SCSG-No mining.

The support resistance should be determined in accordance with the most dangerous situation of roof structure. According to the analysis above, when mining under compaction zone, it results in large periodic weighting. Based on this, establishing roof model of SCSG-No mining as Fig 18.

Where, *ω*_1_ is the subsidence of voussoir beam, m; *θ* is the rotation angle of voussoir beam, °; *h* is the thickness of voussoir beam, m; *P*_0_ is the transmitting load of voussoir beam structure, kN; *L*_1_ is the length of slanting pillar-beam, m; *N*_1_ and *N*_2_ are reaction force on slanting pillar-beam M and N respectively, kN; *m*_1_ is the mining height of upper seam, m; *K*_p_ is bulking coefficient; *G* is the weight of slanting pillar-beam, kN; *α* is caving angle of slanting pillar-beam, °; *q* is uniform distributed reaction force of interburden on upper roof, kN/m.

#### Calculation method of support resistance

Putting the whole of slanting pillar-beam M and N as one object, according to Σ*F*_X_ = 0, Σ*M*_0_ = 0, Σ*M*_F_ = 0, the formulas are listed below:

$$P_{0}\sin\theta +N_{2}\sin\alpha =N_{1}\sin\alpha$$ (1)

$$\frac{2Gm_{1}K_{\text{p}}}{2(K_{\text{p}}-1)\tan\alpha }+N_{1}\left[\frac{m_{1}K_{\text{p}}}{2(K_{\text{p}}-1)\sin\alpha }+L_{1}\cos\alpha \right]=N_{2}\left[\frac{m_{1}K_{\text{p}}}{2(K_{\text{p}}-1)\sin\alpha }-L_{1}\cos\alpha \right]+q2L_{1}\frac{m_{1}K_{\text{p}}}{(K_{\text{p}}-1)\tan\alpha }$$ (2)

$$\frac{2Gm_{1}K_{\text{p}}}{2(K_{\text{p}}-1)\tan\alpha }+N_{1}\left[\frac{m_{1}K_{\text{p}}}{2(K_{\text{p}}-1)\sin\alpha }-L_{1}\cos\alpha \right]+P_{0}\frac{m_{1}K_{\text{p}}\cos(a+\theta )}{(K_{\text{p}}-1)\sin\alpha }=N_{2}\left[\frac{m_{1}K_{\text{p}}}{2(K_{\text{p}}-1)\sin\alpha }-L_{1}\cos\alpha \right]$$ (3)

$$G=bL_{1}\frac{m_{1}K_{\text{p}}}{\left(K_{\text{p}}-1\right)}\rho_{1}g$$ (4)

$$\sin\theta =\frac{\omega_{1}}{2L_{1}}\;\omega_{1}=m_{2}-(K_{\text{p}}-1)\Sigma h$$ (5)

Where, *b* is the width of support, m; *ρ*_1_*g* is the bulk density of slanting pillar-beam, kN/m^3^; *m*_2_ is the mining height of lower coal seam, m; Σ*h* is the thickness of immediate roof, m.

The transmitting load of voussoir beam structure is:

$$P_{0}=b(P_{G}+Q)$$ (6)

$$P_{G}=2hL_{1}\rho g$$ (7)

Where, *P*_G_ is the weight of voussoir beam block, kN/m; *ρg* is the bulk density of voussoir beam, kN/m; The overlying strata form “load-off arch” above the voussoir beam, *Q* is the weight of “load-off arch”, kN/m. According to the Protodyakonov theory [28], *Q* is:

$$Q=\frac{4}{3}\frac{\rho_{1}gL_{1}{}^{2}}{f}$$ (8)

Where, *ρ*_1_g is the bulk density of strata in “load-off arch”, kN/m^3^; *f* is the Protodyakonov coefficient.

The rational support resistance *P* is made up with the weight of immediate roof *W* and transmitting load of slanting pillar-beam structure:

$$P=W+qL_{1}$$ (9)

$$W=bl_{\text{k}}\Sigma h\rho_{1}g$$ (10)

Where, *W* is the weight of immediate roof, kN; *l*_*k*_ is the roof-control distance of support, m; *ρ*_1_*g* is the bulk density of interburden, kN/m^3^.

Based on formulas (1)-(4), *q* can be determined as:

$$q=\frac{bm_{1}K_{\text{p}}\rho_{1}g}{K_{\text{p}}-1}+\frac{P_{0}\cos\alpha \sin(\alpha +\theta )}{L_{1}\sin2\alpha }$$ (11)

Based on formulas (6)-(11), considering supporting efficiency *μ* = 0.9, the rational support resistance of SCSG-No mining is:

$$P_{m}=\frac{1}{\mu }[bl_{k}\Sigma h\rho_{1}g+qL_{1}]$$ (12)

#### Engineering practice

The LW12102 mines No.1-2 seam in Shigetai coal mine, its mining height is 2.8 m and dips 1–3°. The LW101 mines No. 1–2 upper seam, mining height is 2 m, interburden thickness is 4.0 m and *G* is 2.2, therefore, it belongs to SCSG-No mining. The DBT8824/17/35 supports are adopted in LW12102 (Table 2).

**Table 2:** Parameters of supports in LW12102.

Working resistance (kN)	8824
Width (mm)	1750
Support strength (MPa)	0.99–1.03
Opening pressure of safety valve (MPa)	46.2
Support height (mm)	1700–3500
Advancing supports interval (mm)	865
Setting pressure (kN)	5890
Pumping station pressure (MPa)	31.5

Calculating parameters are shown below: *m*_1_ = 2.0 m, *m*_2_ = 2.8 m, Σ*h* = 4 m, *ρ*_1_*g* = 22 kN/m^3^, *L*_1_ = 12 m, *K*_p_ = 1.3, *h* = 5 m, *ρg* = 25 kN/m^3^, *b* = 1.75 m, *l*_*k*_ = 5 m, *α* = 56°, *f* = 4. Combining with formula (12) and calculation parameters above, the rational support resistance is:

$$P_{\text{m}}=\frac{1}{0.9}\times 8323=9247.7\text{kN}$$

According to mining practice of LW12102, the maximum working resistance of No.124 support was 8900 kN, the supports were applied successfully. Therefore, theoretical calculation is basically consistent with engineering practice results.

### Roof structure model of SCSG-S mining

#### Mechanical model of roof structure

**Fig 19:**
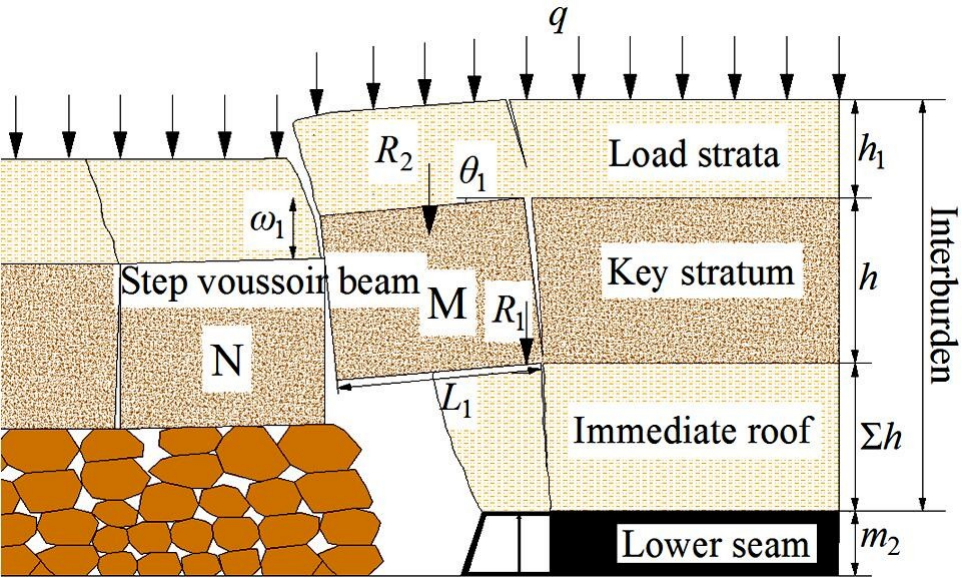
Roof structure model of SCSG-S mining.

Based on the classification, interburden strata can form single key stratum, taking roof structure theory of typical shallow coal seam for reference [2], the single key stratum forms “step voussoir beam structure”, and caving roof of upper coal seam is simplified as uniform distributed load. T he roof structure model with single key stratum is established and shown in Fig 19.

Where, *m*_2_ is the mining height of lower coal seam, m; Σ*h* is the thickness of immediate roof, m; *h* is the thickness of key stratum, m; *h*_1_ is the thickness of load strata, m; *L*_1_ is the length of step voussoir beam block, m; *R*_1_ is the sliding pressure of block M, kN; *R*_2_ is the weight of load strata and block M, kN; *q* is the uniform distributed load, kN/m; *θ*_1_ is rotation angle of block M, °; *ω*_1_ is the subsidence of block M, m.

#### Calculation method of support resistance

The rational support resistance *P* is made up with the weight of immediate roof *W* and the sliding pressure of step voussoir beam structure *R*_1_:

$$P=W+R_{1}$$ (13)

$$W=bl_{\text{k}}\Sigma h\rho_{1}g$$ (14)

According to step voussoir beam theory [2]:

$$R_{1}=[\frac{i-\sin\theta_{1\max}+\sin\theta_{1}-0.5}{i-2\sin\theta_{1\max}+\sin\theta_{1}}]P_{0}$$ (15)

Where, *W* is the weight of immediate roof, kN; *ρ*_1_*g* is the bulk density of immediate roof, kN/m^3^; *P*_0_ is the load of block M and its overlying strata, kN; *i* is lumpiness of step voussoir beam block M; *θ*_1max_ is the maximum rotation angle of block M, °.

*P*_0_ is made up with two parts, one is the weight of block M and load strata *R*_2_, and another is the weight of caving roof of upper coal seam *R*_3_, formula (11) can provide reference for the determination of *q*.

$$P_{0}=R_{2}+R_{3}$$ (16)

$$R_{2}=(h\rho g+h_{1}\rho_{1}g)bL_{1}$$ (17)

$$R_{3}=L_{1}q$$ (18)

Where, *ρg* is the bulk density of step voussoir beam block, kN/m^3^; *ρ*_1_*g* is the bulk density of load strata, kN/m^3^.

Based on formulas (13)-(18), the reasonable support resistance is:

$$P_{\text{m}}=\frac{1}{\mu }\left[bl_{\text{k}}\Sigma h\rho_{1}g+L_{1}\left(\frac{i-\sin\theta_{1\max}+\sin\theta_{1}-0.5}{i-2\sin\theta_{1\max}+\sin\theta_{1}}\right)\cdot (h\rho gb+h_{1}\rho_{1}gb+q)\right]$$ (19)

#### Engineering practice

The LW21305 mines No.1-2 seam in Daliuta coal mine, the mining height is 4.3 m and dips 0–5°, it mines 110–117 m deep, the mining height of No. 1–2 upper seam is 3.8 m, the interburden thickness is 20 m on average and *G* is 4.7, it can form single key stratum, therefore, it belongs to SCSG-S.

Calculating parameters are shown below: *b* = 1.75 m, *l*_k_ = 5.0 m, Σ*h* = 11 m, *ρ*_1_*g* = 23 kN/m^3^, *L*_1_ = 10 m, *h* = 8.4 m, *i =* 0.84, *θ*_1_ = 3°, *θ*_1max_ = 6°, *m*_2_ = 4.3 m, *h*_1_ = 1.0 m, *ρg* = 25 kN/m^3^, *q =* 1377.2 kN/m. Combining with formula (19) and calculation parameters above, the rational support resistance is:

$$P_{\text{m}}=10987.8\text{kN}$$

According to the engineering practice of LW21305, during periodic weighting, working resistance was 10887 kN on average, and the largest working resistance was 11160 kN, the supports were applied successfully. Based on theoretical calculation, the rational support resistance is 10987.8 kN, therefore, it is basically consistent with engineering practice results.

### Roof structure model of SCSG-D mining

#### Mechanical model of roof structure

**Fig 20:**
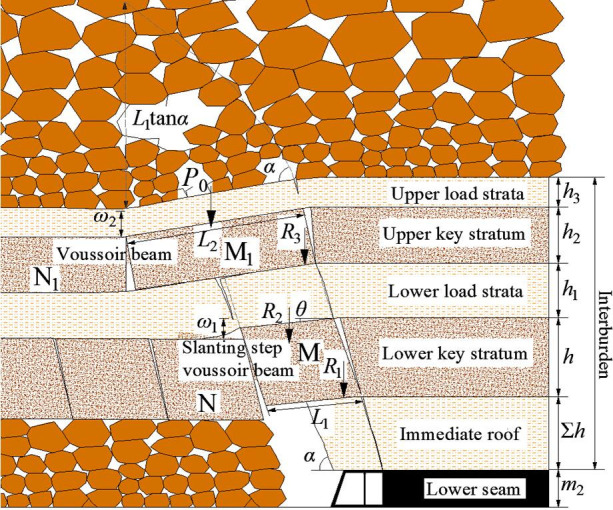
Roof structure model of SCSG-D mining.

According to physical simulation, lower key stratum fractures firstly and forms “slanting step voussoir beam structure”, roof represents small periodic weighting, then, the double key strata fracture simultaneously, and the upper key stratum forms “voussoir beam structure”, roof represents large periodic weighting. Considering the most dangerous situation, the support resistance should be determined by large periodic weighting, its roof structure model is established, shown as Fig 20.

Where, *P*_0_ is the weight of voussoir beam block M_1_ and its overlying strata, kN; *ω*_2_ is the subsidence of voussoir beam block M_1_, m; *L*_2_ is the length of voussoir beam block M_1_, m; *R*_3_ is the sliding pressure of voussoir beam M_1_, kN; *ω*_1_ is the subsidence of block M, m; *R*_2_ is the weight of block M and lower load strata, kN; *θ* is the rotation angle of block M, °; *R*_1_ is the sliding pressure of block M, kN; *L*_1_ is the length of slanting step voussoir beam block M, m; *α* is the caving angle, °; *h*_2_ is the thickness of upper key stratum, m; *h* is the thickness of lower key stratum, m; *h*_3_ is the thickness of upper load strata, m; *h*_1_ is the thickness of lower load strata, m; Σ*h* is the thickness of immediate roof, m; *m*_2_ is the mining height of lower seam, m.

#### Calculation method of support resistance

The rational support resistance *P* is made up with the weight of immediate roof *W* and the sliding pressure of slanting step voussoir beam structure *R*_1_. The upper key stratum, mainly affecting the support resistance through transmitting its load to the lower key stratum structure. Based on this, the support resistance is:

$$P=W+R_{1}$$ (20)

$$W=bl_{\text{k}}\Sigma h\rho_{1}g$$ (21)

According to slanting step voussoir beam theory, *R*_1_ is determined by the formula below:

$$R_{1}=\left[1-\frac{\frac{h}{\sin\alpha }\cos(\alpha -\theta )+\frac{L_{1}}{2}\cos\theta }{\frac{h}{\sin\alpha }\sin(\alpha -\theta )-\omega_{1}-0.5a}\tan\varphi \right]P_{1}$$ (22)

Where, *W* is the weighting of immediate roof, kN; *ρ*_1_*g* is the bulk density of immediate roof, kN/m^3^; *a* is the height of squeezing face, m; *φ* is the friction angle, °; *P*_1_ is the load of slanting step voussoir beam block M, kN.

*P*_1_ is made up with two parts, one is the weight of slanting step voussoir beam block M and lower load strata *R*_2_, and another is transmitting load *R*_3_ by voussoir beam block M_1_.

$$P_{1}=R_{2}+R_{3}$$ (23)

*R*_2_ and *R*_3_ are determined by the following formulas [29]:

$$R_{2}=(h\rho g+h_{1}\rho_{1}g)bL_{1}$$ (24)

$$R_{3}=\left[2+\frac{L_{2}\cot(\varphi +\alpha -\theta )}{2(h_{2}-\omega_{2})}\right]P_{0}$$ (25)

Where, *φ* is the friction angle, °; *ρg* is the bulk density of lower key stratum, kN/m^3^; *ρ*_1_*g* is the bulk density of lower load strata, kN/m^3^.

*P*_0_ is determined by the following formula [30]:

$$P_{0}=bL_{2}h_{2}\rho g+K_{\text{G}}bL_{2}\rho_{1}g\left(h_{3}+\frac{1}{2}L_{2}\tan\alpha \right)$$ (26)

Where, *ρg* is the bulk density of upper key stratum, kN/m^3^; *K*_G_ is load transmitting coefficient; *ρ*_1_*g* is the bulk density of upper load strata and caving roof, kN/m^3^.

According to the previous studies [2], *ω*_1_ ≈ *ω*_2_ = *m*_2_−(*K*_*p*_−1)∑*h*, *K*_p_ = 1.3, *θ* and *a* can be ignored due to the little effect on calculation results. Based on formulas (20)-(26), the rational support resistance of large periodic weighting is:

$$P_{m}=\frac{1}{\mu }\left[bl_{\text{k}}\Sigma h\rho_{1}g+\left(1-\frac{0.5h\cot\alpha +0.25L_{1}}{h-m_{2}+0.3\Sigma h}\right)\cdot \left((h\rho g+h_{1}\rho_{1}g)bL_{1}+2P_{0}+\frac{L_{2}\cot(\varphi +\alpha )}{2(h_{2}-m_{2}+0.3\Sigma h)}P_{0}\right)\right]$$ (27)

#### Engineering practice

The LWN1200 mines No.2-2 seam in Ningtiaota coal mine, the mining height is 5.87 m and it mines 102 m deep on average, the LWN1106 mines the upper No. 1–2 seam, mining height is 1.7 m, the interburden thickness is 39 m on average and *G* is 6.6, therefore, it belongs to SCSG-D.

Calculating parameters are shown below: *b =* 1.75 m, *l*_k_ = 5.0 m, Σ*h* = 5.9 m, *ρ*_1_*g =* 22 kN/m^3^, *h* = 12 m, *α* = 60°, *L*_1_ = 12 m, *m*_2_ = 5.87 m, *h*_1_ = 2.0 m, *ρg* = 25 kN/m^3^, *L*_2_ = 24 m, *φ* = 27°, *h*_2_ = 18 m, *K*_G_ = 0.4, *h*_3_ = 0.8 m. Combining with Formula (27) and calculation parameters above, the rational support resistance is:

$$P_{\text{m}}=13810\;\text{kN}$$

According to engineering practice of LWN1200, during large periodic weighting, the working resistance was 13872 kN, the roof pressure was intense with rib spalling, the supports (Working resistance is 12000 kN) could not satisfy the roof support demand, the rational support resistance by theoretical calculation is 13810kN, therefore, it is also consistent with engineering practice results.

## Conclusions

During shallow coal seam group mining in Western China, the roof weighting is mainly affected by key stratum structure of interburden, and the key stratum structure is closely related to interburden thickness and mining height of lower coal seam. The ratio of interburden thickness to the mining height is defined as a comprehensive index to divide shallow coal seam group, written as *G*.

Based on the key strata in interburden and the index *G*, shallow coal seam group can be divided into three types. The first type is SCSG-No, interburden can not form key stratum structure and shows as immediate roof, *G* is less than 3.3, and roof forms slanting pillar-beam structure. The second type is SCSG-S, *G* is about 4.3–7.3, interburden can form single key stratum. The third type is SCSG-D, *G* is about 5.3–11.3, interburden forms double key strata, the lower key stratum forms slanting step voussoir beam structure, and the upper key stratum forms voussoir beam structure.

SCSG-No, roof weighting is mainly affected by caving roof structure of upper coal seam, when mining under compaction zone, the load of support is the largest. SCSG-S, interburden roof represents step voussoir beam structure. SCSG-D, longwall face has large and small periodic weighting, when the double key strata fracture simultaneously, the roof is the most dangerous. Through establishing the roof structure models, the calculation formulas of support resistance are put forward, and they are verified by engineering practice.
